# Effects of organisational-level interventions at work on employees’ health: a systematic review

**DOI:** 10.1186/1471-2458-14-135

**Published:** 2014-02-08

**Authors:** Diego Montano, Hanno Hoven, Johannes Siegrist

**Affiliations:** 1 Senior Professorship “Work Stress Research”, Faculty of Medicine, Duesseldorf University, Duesseldorf, Germany; 2 Institute of Medical Sociology, Faculty of Medicine, Duesseldorf University, Duesseldorf, Germany

**Keywords:** Occupational health, Employee health, Organisational-level intervention, Effectiveness, Systematic review

## Abstract

**Background:**

Organisational-level workplace interventions are thought to produce more sustainable effects on the health of employees than interventions targeting individual behaviours. However, scientific evidence from intervention studies does not fully support this notion. It is therefore important to explore conditions of positive health effects by systematically reviewing available studies. We set out to evaluate the effectiveness of 39 health-related intervention studies targeting a variety of working conditions.

**Methods:**

Systematic review. Organisational-level workplace interventions aiming at improving employees’ health were identified in electronic databases and manual searches. The appraisal of studies was adapted from the Cochrane Back Review Group guidelines. To improve comparability of the widely varying studies we classified the interventions according to the main approaches towards modifying working conditions. Based on this classification we applied a logistic regression model to estimate significant intervention effects.

**Results:**

39 intervention studies published between 1993 and 2012 were included. In terms of methodology the majority of interventions were of medium quality, and four studies only had a high level of evidence. About half of the studies (19) reported significant effects. There was a marginally significant probability of reporting effects among interventions targeting several organisational-level modifications simultaneously (Odds ratio (OR) 2.71; 95% CI 0.94-11.12), compared to those targeting one dimension only.

**Conclusions:**

Despite the heterogeneity of the 39 organisational-level workplace interventions underlying this review, we were able to compare their effects by applying broad classification categories. Success rates were higher among more comprehensive interventions tackling material, organisational and work-time related conditions simultaneously. To increase the number of successful organisational-level interventions in the future, commonly reported obstacles against the implementation process should be addressed in developing these studies.

## Background

Based on the occupational health principle of “hierarchy of controls” [1] it was proposed that interventions addressing the level of work organisation or work environment may produce more sustainable effects on the health of employees than interventions focusing mainly on individual-level characteristics [2]. This argument is also implicit in influential policy statements and initiatives such as WHO’s Global Strategy for Occupational Health for All (1994) [3], the UK Management Standards [4], or the European Directive 89/391 – OSH, among others [5]. Thus, workplace interventions improving the working conditions are expected to result in substantial reductions of work-related ill health. Such interventions modify specific circumstances under which work is performed [6]; for instance, exposure to physical and chemical agents, working time and intensity, type of employment contract, psychosocial factors at work, work-life balance, and health and safety policies within the organization. Nevertheless, in contrast to rather consistent findings of several meta-analyses of individual-level interventions reporting statistically significant effects on selected health outcomes (e.g. [7-10]), the results of organisational-level interventions have yielded inconsistent findings so far [11-14]. Several systematic reviews have examined the effects of organisational-level interventions on specific health-related outcomes such as work-life balance [15], mental health [16], general health and well-being [17], job stress [2], injury prevention [18], and psychosocial and health effects [19]. In these reviews, a lack of consistency of intervention effects was demonstrated and was discussed in methodological and practical terms. For instance, difficulties of engaging employers, but also employees in intervention activities, interference of the intervention with organisational changes and personnel turnover [20], or inability to adjust for a variety of confounding factors were mentioned [21,22]. Moreover, as employees and (line) managers play an active role in determining how and why interventions work [23] there are potential process-related influences which can hardly be controlled [24]. Understanding how employees appraise the intervention itself, how they are involved in the planning, implementation, and process of interventions, and how line managers’ behaviours may drive or hinder the intervention process are important factors to be taken into account when designing successful worksite intervention studies [23,25,26].

In methodological terms, the paucity and difficulty of implementing organisational-level interventions with highest degree of evidence, i.e. randomized controlled trials (RCT), is one such limiting factor. Even though some authors claim that RCTs are impractical and inadequate in organisational interventions - arguing that they do not take into account psychological and social processes having a causal relationship with the intervention outcomes [24] -, it should be mentioned that interventions as such can be regarded as the distal causes of organisational change and, thus, should be subject to systematic inquiry in terms of RCTs. Another methodological concern of available intervention studies relates to the high degree of heterogeneity of intervention designs, study protocols, intervention measures and study populations. This heterogeneity prevents rapid progress of cumulative knowledge, and it creates obstacles against systematically reviewing available results.

In this contribution we set out to tackle this latter difficulty by evaluating all organisational-level intervention studies identified by our search strategy on the basis of a systematic classification. This classification aims to synthesise the major modifications of the working conditions implemented by the interventions. We distinguish work organisation-directed changes from work time-directed changes and from changes of the material substrate of work (see Methods). By improving the comparability of the reported study findings we aim at identifying the conditions that may explain the effectiveness of interventions at organisational level. To this end, we apply a logistic regression model to estimate significant intervention effects.

## Methods

### Search strategy for the identification of relevant intervention studies

Studies were identified by screening the following databases: ASSIA: Applied Social Sciences Index and Abstracts (SciVerse), Business Source Premier (EBSCO), Cochrane Central Register of Controlled Trials (CENTRAL), Econlit (EBSCO), PubMed (PMC), Scopus (SciVerse), Social Science Citation Index (Web of Knowledge), Sociological Abstracts (ProQuest), and WISO: Wirtschaftswissenschaften. The search was restricted to original papers in peer-reviewed international journals in English, German, French, and Dutch language, published between January 1980 and December 2012. Therefore, neither conference papers or anthologies nor government-commissioned reports were considered. Search terms included various health outcomes, work environment factors, and different intervention methods. The search queries are reported in Additional file 1. The systematic search in databases was amended by search in other systematic reviews, meta-analyses, consulting of experts, and search in relevant websites. Only peer-reviewed studies were assessed. As a quality checklist for reporting the PRISMA statement [27], the Cochrane collaborations tool for risk of bias [28], and the Cochrane Collaboration Back Review Group’s 2003 criteria for deciding quality of evidence [29] were adopted.

Two authors (DM, HH) judged all records on the basis of titles and abstracts. In ambiguous cases papers were discussed and full texts were consulted. In a second step, all selected papers were independently reviewed by the two authors based on full texts, and again, ambiguous cases were discussed. Full analysis of the articles and selection of the studies included in the systematic review were made by the first author.

### Assessment of the risk of bias and level of evidence

As stated in the Introduction, the selection of studies in this systematic review was based on two main criteria: (1) organisational-level interventions at the primary prevention level, and (2) studies aiming to improve health-related outcomes. We did not select specific health outcomes, but included all health measures reported in the studies in order to evaluate a relatively large body of evidence. The assessment of risk of bias and level of evidence was based on the method guidelines for systematic reviews proposed by the Cochrane Collaboration [17,29]. However, we restricted the quality criteria to factors related to the effectiveness of the intervention (some authors labelled these criteria “internal and statistical validity of a study design” [30]). As can be seen from Table 1, each criterion was given equal weight (1 vs. 0), except the criterion ‘sample size’ where we computed quartiles of sample size and assigned weights ranging from 0 (lowest sample sizes) to 3 (highest sample sizes). The reason of emphasizing sample size within the evaluation of study quality is the lack of information on statistical power within the reviewed studies. We therefore used sample size at baseline and at follow-up as proxies of potential statistical power estimates of meeting significant intervention effects. The total quality of each study was defined as the sum of scores obtained in each quality criterion, varying from 0 (lowest) to 12 (highest).

**Table 1:** Quality assessment criteria and weighting scheme

**Criteria**	**Rationale**	**Weights**
Is there a control group?	Reduction of bias of the estimates of intervention effects	0 or 1
Is the baseline response greater than 70%	Reduction of sampling bias	0 or 1
Is the follow-up response greater than 50%	Reduction of attrition and turnover bias	0 or 1
Have the authors adjusted for non-response and drop-out?	Reduction of bias of the estimates of intervention effects by adjusting for attrition and turnover bias	0 or 1
Is there adequate adjustment for the majority of known confounders?	Reduction of bias of the estimates of intervention effects by adjusting for relevant confounders	0 or 1
Where appropriate statistical tests used?	Reduction of bias of the estimates of intervention effects by using appropriate statistical methods	0 or 1
Sample size at baseline. 0 if n is in 1st quantile, 2 if n is in the 2nd quantile, 3 if n is in the 3rd quantile	Validity of statistical inference	0 to 3
Sample size at follow-up. 0 if n is in 1st quantile, 2 if n is in the 2nd quantile, 3 if n is in the 3rd quantile.	Validity of statistical inference	0 to 3

As an additional relevant criterion of evaluation, the level of evidence was assessed by taking into account the study design (Table 2). By level of evidence we mean the extent to which a study is capable of detecting and estimating a real intervention effect with minimum bias. In order to simplify the presentation of results, three levels of evidence only were defined. The highest level of evidence in the context of our systematic review was assumed for randomized controlled trials (RCT) whose quality score – as defined before – had to meet a level above the median of the quality scores of all studies. Given the fact that randomisation minimises the risk of bias in comparison with all other designs [31], this type of study design is likely to produce the relatively strongest degree of evidence of an intervention effect [32]. However, given the difficulties of RCTs discussed above, quasi-experimental designs are generally applied more often in this field of research. If they contain well-defined control groups [33], quasi-experimental studies meeting the defined quality standard are judged as meeting a medium level of evidence. They share this level with those RCT studies that failed to reach the defined quality standard. All remaining studies were classified as belonging to a low category of evidence, being vulnerable to confounding bias [30,34].

**Table 2:** Scheme of level of evidence for each single study

**Level**	**Definition**
High	The study has a randomized controlled design whose quality is greater than the median study quality.
Medium	The study is either a randomized controlled design whose quality is less than the median study quality, or the study is a quasi-experimental control study whose quality is greater than the median study quality
Low	The study is either a quasi-experimental control trial whose quality is less than the median study quality, or the study is a quasi-experimental one-group prospective or a cross-sectional one.

### Classification of the major modifications of the working conditions implemented by the interventions

Given the complexity and variety of implementations in the interventions we proposed three broad categories, referring to a classification of conditions of work used in the European Working Conditions Survey [35,36].

1. Material conditions. All sorts of physical and chemical agents implied in the execution of work tasks are included (e.g. vibrations, noise, chemical substances, ergonomics).

2. Work time-related conditions. These conditions relate to the amount of working time and intensity of work, the latter being measured as number of activities per time unit (e.g. work speed, shifts, deadlines, pace of work, breaks).

3. Work organisation conditions. These include a variety of psychological and social factors (job demands, job control, efforts and rewards, responsibility, etc.), and processes and procedures required to accomplish work tasks (e.g. methods of work, order of tasks, team organization, structure of hierarchy, security guidelines training).

This classification is not exclusive as studies may address more than one intervention target simultaneously. In fact, it is our aim to explore to what extent a more comprehensive intervention is better suited to produce significant health effects, compared to interventions that are restricted to one single target (see below). We exemplify our classification procedure by illustrating one case in more detail [37]. The purpose of the intervention of Morken and colleagues was to enable operators and the organisation to prevent and cope with musculoskeletal problems at the workplace. The intervention consisted of ten didactic sessions during which operators found solutions on how to obtain an optimal work environment both organisationally and technically. Some of the changes implemented during the intervention period were redesigning the workplace, reducing repetitive-motion stress points and modifying the work process to promote job variations. According to our classification scheme, these changes can be classified as changes of the material and organisational conditions of work since they include both ergonomic and work process changes. It should be noted that by roughly merging our classification of intervention targets with the classification of the European Working Conditions Survey, we intend to advance the comparison of results of intervention studies with results of surveys, thus strengthening cross-fertilization and policy impact of the two research strands (see e.g. [38]).

### Estimation of the probability of reporting significant intervention effects

In order to evaluate to what extent modification of the working conditions may be associated with the reporting of statistically significant effects we estimated a logistic regression model. To this end, we define the reporting of significant intervention effects (which we consider a proxy of intervention effectiveness) as dependent variable. Clearly, the heterogeneity of study designs and of outcome variables does not allow a meta-analysis. We therefore restrict our quantitative analysis to the test of a research hypothesis. Our hypothesis claims that the probability of reporting statistically significant effects increases with an increased degree of comprehensiveness of intervention targets. To test this hypothesis a logistic regression model was used to estimate the probability of reporting positive intervention effects as a function of the number of working conditions changed by the intervention.

There are at least two major confounders which have to be addressed when testing the hypothesis. First, it may be that studies addressing several intervention targets simultaneously score higher in terms of study quality, including study design. Therefore, we control for this effect by appropriate adjustment in the regression model. Second, since the socioeconomic characteristics of the samples define a particular work environment and social setting that usually influences the intervention effectiveness [39,40], we control for this effect by classifying the study samples according to an internationally established classification of occupational positions, the Erikson-Goldthorp-Portocarrero scheme (EGP) [41]. Due to the lack of specific information regarding the occupations of the employees in some of the studies, two major groups of occupations only could be broadly identified:

1. *EGP classes I to III*. Higher and lower service occupations, routine clerical and sales occupations (e.g. managers, professionals, routine clerical and sales workers).

2. *EGP classes VI to VII*. Skilled manual and semi- or unskilled manual occupations (e.g. craft workers, skilled service, skilled machine operations, and elementary labourers).

As logistic regression models overestimate the odds ratios in small sample sizes [42], odds ratios were estimated by penalized maximum likelihood and hypothesis testing by likelihood-ratio tests [43,44]. All analyses were conducted with the programming language R.

## Results

The search in the databases and the manual search resulted in 18 145 initial records after checking for duplicates. Based on titles and abstracts 699 studies were submitted to further appraisal. Applying the selection criteria mentioned in the Methods Section 77 studies were selected for detailed analysis. Of these, 39 intervention studies meeting fully the criteria were included in the systematic review. A detailed description of main characteristics of the studies and their implementation is reproduced in Additional file 2: Tables S1 and S2.

A brief overview of the main characteristics of the studies is reproduced in Table 3. The majority of studies followed a participatory approach, and many investigations applied a quasi-experimental prospective design with a control group. The median of follow-up time was one year. About half of the studies reported statistically significant intervention effects on health-related outcomes. Four studies were considered as having a high level of evidence. In a majority of studies employees belonged to occupational classes I to III. Only six studies included all occupational classes. Studies were conducted mostly in urban areas and targeted usually health care workers (at least 14 studies), manufacture workers (at least four studies) and civil servants (at least three studies). Sample sizes at baseline varied considerably in the range of 41 to 3506 participants, even though 28 out of 39 studies had sample sizes less than 500 employees.

**Table 3:** Descriptive statistics of studies reviewed

**Variable**	**Number of studies/median and range**
***EGP occupational class***	
I-III	18
I-III, VI-VII	6
VI-VII	15
***Study design***	
Quasi-experimental, cross-sectional	1
Quasi-experimental one-group prospective	9
Quasi-experimental prospective with control group	21
Quasi-experimental prospective and retrospective	2
Randomized controlled	6
***Evidence grade***	
High	4
Medium	25
Low	10
***Significant intervention effects***	
No	21
Yes	18
***Intervention type***	
Organisational intervention (OI)	9
Participatory (including Participatory Ergonomics PE and Participatory Research Action PAR)	7
Shift schedules	18
Other	5
Year of publication	2005 [1993, 2012]
Sample size at baseline	300 [41, 3506]
Sample size at follow-up	187 [36, 2617]
Sample size of control group	122 [31, 347]
Follow-up time (years)	1 [0.25, 13]
Study quality	7 [1,11]
Number of working conditions changed	2 [1,4]

An analysis of the frequencies of specific outcomes showed that interventions focused mostly on burnout (six studies), absenteeism (four studies), musculoskeletal disorders of the upper body (six studies), and depressive symptoms (three studies). The studies were conducted in the following industrialized countries: Australia, Canada, Italy, Germany, the Scandinavian countries, USA, Japan, UK and The Netherlands.

In Table 4 the frequencies of reporting statistically significant effects for each category of working conditions are reported. Ten studies introduced changes in the material and organisational conditions, 16 studies concentrated on organisational conditions, whereas eight studies emphasised on the time conditions of work. Three studies comprised all three types of working conditions and reported statistically significant effects on burnout and injury prevention [45-47]. The changes implemented by these interventions included staffing processes, work reorganisation, training, ergonomics, assessment of factors relating to iterative injuries, and control processes.

**Table 4:** Types of modified working conditions and frequency of significant intervention effects

		**Statistically significant?**	
**Working condition**	**Number of modified working conditions**	**No**	**Yes**
Material	1	1	1
Material, organisation	2	4	6
Material, time, organisation	3	0	3
Organisation	1	10	6
Time	1	3	2
Time, organisation	2	2	1

At the same time, interventions modifying material and organisational conditions of work also report more frequently significant effects (six out of ten studies). These interventions involved, among others, the introduction of mechanical lifting equipment and employee training [48], improvements of machine performance and communication between workers and supervisors [49], reduction of lifting loads and rotation schedules [50], increased compliance with the use of protective substances and establishment of improved control procedures of occupational risks [51], improvement of technical equipment and health surveillance [52], as well as substituting chemicals and increased safety guidelines in the organisation [53]. The health-related outcomes of these interventions included back pain, injuries, sick leave, blood pressure, and eczema.

If the results of each intervention study are compared according to their level of evidence, three out of four high-evidence studies did not report significant intervention effects [37,54,55], whereas 14 out of 25 medium evidence level studies reported significant improvements of health-related outcomes, such as ischemic heart disease risk [56], burnout [45,46,57,58], lost time injury [47], perceived health [59], blood pressure [60], decreased mental distress and better sleep [61], reduction of sick-leave length [49], back-pain related lost working days [50], eczema incidence [53], and mental health [62]. Finally, four out of ten low evidence studies reported statistically significant changes in outcomes such as self-rated health [63] and injury rates [48,52,64].

Concerning our hypothesis of an association between the probability of reporting statistically significant effects and the number of modified working conditions, results of a respective logistic regression model are given in Table 5. This analysis takes into account the potential impact of occupational class of employees as well as the quality of the study. As can be seen, the number of working conditions modified by the intervention tends to be associated with increased intervention success (even though only marginally significant, OR = 2.71, 95% CI 0.94 – 11.12). In line with the findings given in Table 4, it seems that interventions addressing different types of working conditions simultaneously may increase the chance of producing a beneficial effect on the outcomes under study. When interpreting the result, it should be kept in mind that two of the three papers analysing comprehensive interventions result from the same intervention study, exploring health outcomes after one year [45] and after three years [46].

**Table 5:** **Logistic regression model estimated by penalized maximum likelihood**^**a**^

	**OR**	**Lower 95% CI**	**Upper 95% CI**	**P**
Number of modified working conditions	2.71	0.94	11.12	0.07
Study quality	0.95	0.75	1.18	0.63
**EGP class**				
Reference I-III				
I-III, VI-VII	0.42	0.05	2.66	0.37
VI-VII	0.73	0.14	3.14	0.68

^a^Dependent variable: reporting of significant effects (coded yes/no). Working conditions and study quality treated as metric variables. Pseudo R^2^ = 0.12.

## Discussion

In this systematic review 39 organisational-level workplace interventions were analysed with regard to their effects on employee health. We tackled the large heterogeneity of study designs, intervention targets and health outcomes by classifying the studies according to three general categories of intervention targets (material, organisational, work time-related). About half of the studies yielded statistically significant intervention effects. Favourable health outcomes were reported for self-rated mental and general health, and for reduction of injury rates. Taking the potential impact of study quality and occupational class into account, our main finding revealed that more comprehensive interventions addressing several organisational-level targets simultaneously had a higher chance of reporting significant health improvements than those restricted to one intervention target. Before discussing limitations and strengths of this study the following questions are addressed. First, how can we explain the relatively low success rate of these interventions? And second, how can we interpret the finding that more comprehensive interventions are more likely to result in positive health outcomes among employees?

The success rate of the 39 interventions reviewed in this study is substantially lower than the one reported in an important earlier systematic review by LaMontagne et al. [2] where about two thirds of the 18 intervention studies demonstrated a significant effect. It seems particularly worrying that three of the four studies with highest level of evidence in our review failed to achieve a positive outcome [37,54,55]). Therefore, it is instructive to learn how the authors themselves explained the limited success of their interventions. In a synthesis of their arguments the following reasons can be distinguished. First, in some cases there was not sufficient participation of employees in preparing the intervention, or there was a lack of communication and motivation to support the intervention and to comply with organisational changes [65-68]. Second, the implementation of some interventions was difficult because some aspects did not work as anticipated or could not be realised as originally designed [39,55].Third, there were difficulties in developing or maintaining the intervention due to lack of support from employers and managers [62,66,69]. Fourth, the intervention was threatened by external events or conditions that were beyond the control of the responsible team (e.g. job turnover, organisational restructuring or merging) [52,53,70]. Finally, the lack of significant effects was eventually due to a short follow-up time, to a weak treatment effect, or to a failure of controlling for potential confounders of intervention effects [21,58,65,68,69,71]. These reasons clearly point to the need of developing interventions in a participatory approach, where “organizational interventions (are) to be understood as a collective of initiatives and change activities, competing and intertwining with a multitude of concurrent events” [72]. Moreover, careful process evaluation should be a mandatory part of any intervention trial.

How can we interpret the finding that more comprehensive interventions are more likely to result in positive health outcomes among employees? It is of interest to note that individual-level stress management interventions demonstrated exactly the opposite trend. Effect sizes were strongest if the treatment focused on cognitive behavioural therapy or on an intervention that strengthened employees’ resources of coping with stressful work, whereas the effects of multimodal interventions were much weaker [9]. In case of organisational-level interventions, the situation is different. Given the many difficulties of implementation, including the interference of a structural change with established organisational procedures, it may be crucial to develop a comprehensive set of modifications that can exert sufficient impact on employees who are exposed to a complex work environment. This is, for instance, the case if material improvements of work tasks are linked with changes in the division of work or other features of work organisation, and/or with increased flexibility of work schedules. Thus, the combination of a hierarchy of controls approach with specific improvements of technical, chemical or physical elements of the work environment may contribute to a reduction of risk exposure and related adverse health effects.

### Limitations

This systematic review has several limitations. First, the inconsistency of health effects of interventions regarding changes of the working conditions should be interpreted with caution, since study designs, outcomes and corresponding measures are highly heterogeneous. Second, it is possible that we bypassed some relevant intervention studies during our search strategy, in particular as we did not include intervention studies published in books, anthologies, working papers or other grey literature sources. Third, neither the weighting scheme of the study quality nor the classification of working conditions is based on internationally adopted procedures. The broad classification of working conditions may obscure potential significant effects of specific intervention designs within each category. For instance, if we had classified all studies based on a distinct intervention design (e.g. job-demand-control model [73]) separately, i.e. independent of the three intervention approaches underlying our systematic review, different frequencies of successful interventions could have resulted. As an alternative classification, the presence or absence of an explicit theoretical basis of intervention could be applied, especially so in the area of a health-adverse psychosocial work environment. For instance, many respective interventions were based on the job-demand-control model. Yet, success rates were not significantly higher, probably due to the difficulties of implementation discussed above. Fourth, the scope of occupations exposed to organisational-level interventions was limited, and a substantial part of the studies under review was addressed to health care workers (15 studies or about 38%), thus limiting the generalisation of results. Finally, it cannot be ruled out that some intervention effects are actually due to regression to the mean which may overestimate the intervention effects.

These limitations are balanced by several strengths. First, we collected a relatively large number of intervention studies from different countries, addressing modifications at the organisational level to different occupational groups and exploring different health outcomes. By doing so we minimised a selection bias of results answering our main research question. Second, we tackled the challenge of diversity of study designs and intervention targets by classifying the studies into categories amenable to comparative analysis. Classification schemes concerned the quality of studies and the type of organisational-level interventions. Third, this procedure enabled us to estimate which factors were likely to produce favourable health outcomes among employees. More specifically, we tested the hypothesis that this was the case if interventions were designed as comprehensive modifications, combining material, organisational, and work time-related improvements.

## Conclusions

Despite the heterogeneity of the 39 organisational-level workplace interventions underlying this review, we were able to compare their effects by applying broad classification categories. About half of the studies reveal significant effects on employees’ health. Success rates were higher among more comprehensive interventions tackling material, organisational and work-time related conditions simultaneously. To increase the number of successful organisational-level interventions in the future commonly reported obstacles against the implementation process should be addressed in developing these studies.

## Competing interests

The authors declare that they have no competing interests.

## Authors’ contributions

DM contributed substantially to the search strategy, study appraisal, statistical analyses and manuscript preparation. HH contributed to the development of the search strategy and collaborated in performing the study appraisal and manuscript preparation. JS led the drafting of the manuscript and prepared the final version. All authors read and approved the final manuscript.

## Pre-publication history

The pre-publication history for this paper can be accessed here:

http://www.biomedcentral.com/1471-2458/14/135/prepub

## Supplementary Material

Additional file 1 Search Queries. Click here for file

Additional file 2 Results Tables. Click here for file
