# Supplementary material for: Effects of organisational-level interventions at work on employees’ health: a systematic review
Source: BMC Public Health. 2014 Feb 8;14:135. doi: 10.1186/1471-2458-14-135 (PMC3929163; doi:10.1186/1471-2458-14-135)
Supplement: Additional file 1 — Search Queries. [file 1471-2458-14-135-S1.doc]

# Effects of organisational-level interventions at work on employees’ health: A systematic review

Diego Montano^1*^, Hanno Hoven^1,2^, Johannes Siegrist^1^

*^1^ Senior Professorship “Work Stress Research”, Faculty of Medicine, Duesseldorf University*

*^2^Institute of Medical Sociology, Faculty of Medicine, Duesseldorf University*

*Correspondence: [diego.montano@med.uni-duesseldorf.de](mailto:diego.montano@med.uni-duesseldorf.de)

# Appendix 1. Search queries

# PubMed (PMC)

((“health”[All Fields] OR (“subjective”[All Fields] AND "health"[All Fields]) OR (“health”[All Fields] AND ("manpower"[All Fields] OR "worker*"[All Fields])) OR ("physical"[All Fields] AND "health"[All Fields]) OR ("mental"[All Fields] AND “health"[All Fields]) OR ("general"[All Fields] AND "health"[All Fields]) OR ("functioning"[All Fields] AND "health"[All Fields]) OR functioning[All Fields] OR functional limitations[All Fields] OR "absenteeism"[All Fields] OR (“sickness”[All Fields] AND “absence”[All Fields]) OR ("depressive"[All Fields] AND "disorder"[All Fields]) OR ("affective"[All Fields] AND "disorder"[All Fields]) OR depression[All Fields] OR “disease*”[All Fields] OR cardiovascular[All Fields] OR ("cardiovascular"[All Fields] AND “disease*”[All Fields]) OR ("heart"[All Fields] AND “disease*”[All Fields]) OR coronary[All Fields] OR "stroke"[All Fields] OR (ischaemia[All Fields] OR "ischemia"[All Fields] OR “ischem*”[All Fields]) OR myocard*[All Fields] OR "hypertension"[All Fields] OR "obesity"[All Fields] OR diabetes[All Fields] OR "overweight"[All Fields] OR "cholesterol"[All Fields] OR musculoskeletal[All Fields] OR (“musculoskeletal”[All Fields] AND “disorder”[All Fields]) OR "blood pressure"[All Fields] OR ("back"[All Fields] AND "pain"[All Fields]) OR “disability”[All Fields] OR (“wound*”[All Fields] AND “injur*”[All Fields]) OR "injur*"[All Fields] OR "wounds"[All Fields] OR (“work”[All Fields] AND ("accidents"[All Fields] OR “accidents”[MeSH Terms])) OR morbidity[All Fields] OR mortality[All Fields] OR burnout[All Fields] OR (“all-cause”[All Fields] AND "mortality"[All Fields]))

AND ((“psychosocial”[All Fields] AND ("stress"[All Fields] OR “stressors”[All Fields] OR "risk"[All Fields] OR "conditions"[All Fields])) OR (“psychological”[All Fields] AND ("stress"[All Fields] OR “stressors”[All Fields])) OR "demand-control"[All Fields] OR (“support”[All Fields] AND work[All Fields]) OR (“demand”[All Fields] AND "control"[All Fields]) OR (“effort”[All Fields] AND "reward"[All Fields]) OR (“effort-reward”[All Fields] AND “imbalance”[All Fields]) OR ("organizational"[All Fields] AND "justice"[All Fields]) OR ("organisational"[All Fields] AND "justice"[All Fields]) OR (“strain”[All Fields] AND (work[All Fields] OR job[All Fields])) OR (“job”[All Fields] AND “task”[All Fields] AND “control”[All Fields]) OR ("work*"[ All Fields] AND “conditions”[All Fields]) OR “psychosocial”[All Fields] OR "workplace"[All Fields] OR “job*”[All Fields] OR “downsizing”[All Fields] OR “overtime”[All Fields] OR “ergonomic”[All Fields] OR ((“physical”[All Fields] OR “chemical”[All Fields]) AND “hazard*”[All Fields]) OR ((“night”[All Fields] OR “day”[All Fields]) AND “work”[All Fields]) OR (“occupation*”[All Fields] AND “stress*”[All Fields]) OR (“work*”[All Fields] AND “characteristics”[All Fields]))

AND (intervention[Title/Abstract])

AND (“management”[All Fields] OR "organization"[All Fields] OR "organisation"[All Fields] OR ("disease"[All Fields] AND "management"[All Fields]) OR ("health"[All Fields] AND “circles”[All Fields]) OR “amigo”[All Fields] OR (“primary”[All Fields] AND "intervention"[All Fields]) OR “prima-ef”[All Fields] OR ("health"[All Fields] AND "management"[All Fields]) OR (“return to”[All Fields] AND “work”[All Fields]) OR (“return-to”[All Fields] AND “work”[All Fields]) OR ("workplace"[All Fields] AND "health"[All Fields]) OR ("health"[All Fields] AND "promotion"[All Fields]) OR (“shift”[All Fields] AND "work"[All Fields]) OR (“psychosocial”[All Fields] AND "risk"[All Fields]) OR "risk management"[All Fields] OR “self-scheduling”[All Fields] OR (“flexible”[All Fields] AND “scheduling”[All Fields]) OR (“work”[All Fields] AND “schedule*”[All Fields]) OR (“flexible”[All Fields] AND "work"[All Fields]) OR (“compressed”[All Fields] AND (“hour”[All Fields] OR "work"[All Fields])) OR (“compressed”[All Fields] AND “week”[All Fields]) OR (“flexible”[All Fields] AND “salary”[All Fields]) OR ("life"[All Fields] AND “balance"[All Fields]) OR (“work”[All Fields] AND "life"[All Fields] AND “balance"[All Fields]) OR ("life"[All Fields] AND “family"[All Fields]) OR ("reconciling"[All Fields] AND “work"[All Fields]) OR “employee*”[All Fields] OR “employer*”[All Fields] OR ("quality"[All Fields] AND "life"[All Fields]) OR "quality of life"[All Fields] OR ((“cognitive”[All Fields] AND “therapy”[All Fields]) AND “behavi*”[All Fields])))

AND ("1980/01/01"[Date - Publication]: "3000"[Date - Publication])

# Scopus (SciVerse)

(TITLE-ABS-KEY(health AND (subjective OR mental OR physical OR general)) OR TITLE-ABS-KEY(manpower) OR TITLE-ABS-KEY(worker) OR TITLE-ABS-KEY(functioning AND health) OR TITLE-ABS-KEY(functioning) OR TITLE-ABS-KEY(functional AND limitations) OR TITLE-ABS-KEY(absenteeism) OR TITLE-ABS-KEY(sickness AND absence) OR TITLE-ABS-KEY(depressive AND disorder) OR TITLE-ABS-KEY(affective AND disorder) OR TITLE-ABS-KEY(depression) OR TITLE-ABS-KEY(disease) OR TITLE-ABS-KEY(cardiovascular AND disease*) OR TITLE-ABS-KEY(heart AND disease) OR TITLE-ABS-KEY(coronary) OR TITLE-ABS-KEY(stroke) OR TITLE-ABS-KEY(ischem*) OR TITLE-ABS-KEY(ischaemia) OR TITLE-ABS-KEY(myocard*) OR TITLE-ABS-KEY(hypertension) OR TITLE-ABS-KEY(obesity) OR TITLE-ABS-KEY(diabetes) OR TITLE-ABS-KEY(overweight) OR TITLE-ABS-KEY(cholesterol) OR TITLE-ABS-KEY(musculoskeletal AND disorder) OR TITLE-ABS-KEY(blood AND pressure) OR TITLE-ABS-KEY(back AND pain) OR TITLE-ABS-KEY(disability) OR TITLE-ABS-KEY(wound) OR TITLE-ABS-KEY(injur*) OR TITLE-ABS-KEY(work AND accidents) OR TITLE-ABS-KEY(accidents) OR TITLE-ABS-KEY(morbidity) OR TITLE-ABS-KEY(mortality) OR TITLE-ABS-KEY(burnout) OR TITLE-ABS-KEY(all-cause AND mortality))

AND (TITLE-ABS-KEY(psychosocial AND (stress OR stressors OR conditions OR risk)) OR TITLE-ABS-KEY(psychological AND (stress OR stressors)) OR TITLE-ABS-KEY(demand-control) OR TITLE-ABS-KEY(support AND work) OR TITLE-ABS-KEY(work AND control) OR TITLE-ABS-KEY(effort AND reward) OR TITLE-ABS-KEY(effort-reward AND imbalance) OR TITLE-ABS-KEY(organizational AND justice) OR TITLE-ABS-KEY(organisational AND justice) OR TITLE-ABS-KEY(strain AND (work AND job)) OR TITLE-ABS-KEY(work* AND conditions) OR TITLE-ABS-KEY(psychosocial) OR TITLE-ABS-KEY(workplace) OR TITLE-ABS-KEY(job*) OR TITLE-ABS-KEY(downsizing) OR TITLE-ABS-KEY(overtime) OR TITLE-ABS-KEY(ergonomic) OR TITLE-ABS-KEY((physical OR chemical) AND hazards) OR TITLE-ABS-KEY((night OR day) AND work) OR TITLE-ABS-KEY(occupation* AND stress) OR TITLE-ABS-KEY(work* AND characteristics)) AND (TITLE-ABS-KEY(intervention*)) AND (TITLE-ABS-KEY(work*))

AND (TITLE-ABS-KEY(disease AND management) OR TITLE-ABS-KEY(health AND circles) OR TITLE-ABS-KEY(amigo) OR TITLE-ABS-KEY(primary AND intervention) OR TITLE-ABS-KEY(prima-ef) OR TITLE-ABS-KEY(health AND management) OR TITLE-ABS-KEY(workplace AND health) OR TITLE-ABS-KEY(health AND promotion) OR TITLE-ABS-KEY(shift AND work) OR TITLE-ABS-KEY(psychosocial AND risk) OR TITLE-ABS-KEY(risk management) OR TITLE-ABS-KEY(self-scheduling) OR TITLE-ABS-KEY(flexible AND scheduling) OR TITLE-ABS-KEY(work AND schedule*) OR TITLE-ABS-KEY(reconciling AND work) OR TITLE-ABS-KEY(family AND life) OR TITLE-ABS-KEY(return to AND work) OR TITLE-ABS-KEY(return-to AND work) OR TITLE-ABS-KEY(flexible AND work) OR TITLE-ABS-KEY(flexibility) OR TITLE-ABS-KEY(compressed AND (hour OR work)) OR TITLE-ABS-KEY(compressed AND week) OR TITLE-ABS-KEY(flexible AND salary) OR TITLE-ABS-KEY(life AND balance) TITLE-ABS-KEY(work AND life AND balance) OR TITLE-ABS-KEY(employee*) OR TITLE-ABS-KEY(employer*) OR TITLE-ABS-KEY(quality AND life) OR TITLE-ABS-KEY(quality of life) OR TITLE-ABS-KEY(cognitive AND (therapy OR behav*)))

AND (PUBYEAR > 1980)

# ASSIA and Sociological Abstracts (ProQuest)

(ti,ab((health AND (subjective OR mental OR physical OR general)) OR manpower OR worker OR (functioning AND health) OR functioning OR (functional AND limitations) OR absenteeism OR (sickness AND absence) OR (depressive AND disorder) OR (affective AND disorder) OR depression OR disease OR (cardiovascular AND disease) OR (heart AND disease) OR coronary OR stroke OR ischem OR ischaemia OR myocard OR hypertension OR obesity OR diabetes OR overweight OR cholesterol OR (musculoskeletal AND disorder) OR musculoskeletal OR (blood AND pressure) OR (back AND pain) OR disability OR wound OR injury OR (work AND accidents) OR accidents OR morbidity OR mortality OR burnout OR (all-cause AND mortality)))

AND (ti,ab((psychosocial AND (stress OR stressors OR conditions OR risk)) OR (psychological AND (stress OR stressors)) OR demand-control OR (support AND work) OR (work AND control) OR (effort AND reward) OR (effort-reward AND imbalance) OR (organizational AND justice) OR (organisational AND justice) OR (strain AND (work AND job)) OR (work AND conditions) OR psychosocial OR workplace OR job OR downsizing OR overtime OR ergonomic OR (hazard AND (physical AND chemical)) OR (work AND (night OR day)) OR (occupation AND stress) OR (work AND characteristics)))

AND (ti,ab((disease AND management) OR (health AND circles) OR amigo OR (primary AND intervention) OR prima-ef OR (health AND management) OR (workplace AND health) OR (health AND promotion) OR (shift AND work) OR risk management OR self-scheduling OR (flexible AND scheduling) OR (work AND schedule) OR (reconciling AND work) OR (family AND life) OR (return-to AND work) OR (flexible AND work) OR flexibility OR (compressed AND (hour OR work OR week)) OR (flexible AND salary) OR (life AND balance) OR (work AND (life OR balance)) OR employee OR employer OR (quality AND life) OR (cognitive AND (therapy OR behavi*))))

AND (ti,ab(intervention*)) AND yr(>1979)

# Business Source Premier and Econlit (EBSCO)

(TX (health AND (subjective OR mental OR physical OR general)) OR manpower OR worker OR (functioning AND health) OR functioning OR (functional AND limitations) OR absenteeism OR (sickness AND absence) OR (depressive AND disorder) OR (affective AND disorder) OR depression OR disease OR (cardiovascular AND disease) OR (heart AND disease) OR coronary OR stroke OR ischem OR ischaemia OR myocard OR hypertension OR obesity OR diabetes OR overweight OR cholesterol OR (musculoskeletal AND disorder) OR musculoskeletal OR (blood AND pressure) OR (back AND pain) OR disability OR wound OR injury OR (work AND accidents) OR accidents OR morbidity OR mortality OR burnout OR (all-cause AND mortality))

AND (TX (psychosocial AND (stress OR stressors OR conditions OR risk)) OR (psychological AND (stress OR stressors)) OR demand-control OR (support AND work) OR (work AND control) OR (effort AND reward) OR (effort-reward AND imbalance) OR (organizational AND justice) OR (organisational AND justice) OR (strain AND (work AND job)) OR (work AND conditions) OR psychosocial OR workplace OR job OR downsizing OR overtime OR ergonomic OR (hazard AND (physical AND chemical)) OR (work AND (night OR day)) OR (occupation AND stress) OR (work AND characteristics))

AND (TX (disease AND management) OR (health AND circles) OR amigo OR (primary AND intervention) OR prima-ef OR (health AND management) OR (workplace AND health) OR (health AND promotion) OR (shift AND work) OR risk management OR self-scheduling OR (flexible AND scheduling) OR (work AND schedule) OR (reconciling AND work) OR (family AND life) OR (return-to AND work) OR (flexible AND work) OR flexibility OR (compressed AND (hour OR work OR week)) OR (flexible AND salary) OR (life AND balance) OR (work AND (life OR balance)) OR employee OR employer OR (quality AND life) OR (cognitive AND (therapy OR behavio#ral)))

AND ((TI intervention*) OR (AB intervention*))

# Social Science Citation Index (Web of Knowledge)

(TS=((health AND (subjective OR mental OR physical OR general)) OR manpower OR worker OR (functioning AND health) OR functioning OR (functional AND limitations) OR absenteeism OR (sickness AND absence) OR (depressive AND disorder) OR (affective AND disorder) OR depression OR disease OR (cardiovascular AND disease) OR (heart AND disease) OR coronary OR stroke OR ischem OR ischaemia OR myocard OR hypertension OR obesity OR diabetes OR overweight OR cholesterol OR (musculoskeletal AND disorder) OR musculoskeletal OR (blood AND pressure) OR (back AND pain) OR disability OR wound OR injury OR (work AND accidents) OR accidents OR morbidity OR mortality OR burnout OR (all-cause AND mortality)))

AND (TS=((psychosocial AND (stress OR stressors OR conditions OR risk)) OR (psychological AND (stress OR stressors)) OR demand-control OR (support AND work) OR (work AND control) OR (effort AND reward) OR (effort-reward AND imbalance) OR (organizational AND justice) OR (organisational AND justice) OR (strain AND (work AND job)) OR (work AND conditions) OR psychosocial OR workplace OR job OR downsizing OR overtime OR ergonomic OR (hazard AND (physical AND chemical)) OR (work AND (night OR day)) OR (occupation AND stress) OR (work AND characteristics)))

AND (TS=((disease AND management) OR (health AND circles) OR amigo OR (primary AND intervention) OR prima-ef OR (health AND management) OR (workplace AND health) OR (health AND promotion) OR (shift AND work) OR risk management OR self-scheduling OR (flexible AND scheduling) OR (work AND schedule) OR (reconciling AND work) OR (family AND life) OR (return-to AND work) OR (flexible AND work) OR flexibility OR (compressed AND (hour OR work OR week)) OR (flexible AND salary) OR (life AND balance) OR (work AND (life OR balance)) OR employee OR employer OR (quality AND life) OR (cognitive AND (therapy OR behavi*))))

AND (TI=(intervention*))

# Cochrane (Wiley)

((health AND (subjective OR mental OR physical OR general)) OR manpower OR worker OR (functioning AND health) OR functioning OR (functional AND limitations) OR absenteeism OR (sickness AND absence) OR (depressive AND disorder) OR (affective AND disorder) OR depression OR disease OR (cardiovascular AND disease) OR (heart AND disease) OR coronary OR stroke OR ischem OR ischaemia OR myocard OR hypertension OR obesity OR diabetes OR overweight OR cholesterol OR (musculoskeletal AND disorder) OR musculoskeletal OR (blood AND pressure) OR (back AND pain) OR disability OR wound OR injury OR (work AND accidents) OR accidents OR morbidity OR mortality OR burnout OR (all-cause AND mortality))

AND ((psychosocial AND (stress OR stressors OR conditions OR risk)) OR (psychological AND (stress OR stressors)) OR demand-control OR (support AND work) OR (work AND control) OR (effort AND reward) OR (effort-reward AND imbalance) OR (organizational AND justice) OR (organisational AND justice) OR (strain AND (work AND job)) OR (work AND conditions) OR psychosocial OR workplace OR job OR downsizing OR overtime OR ergonomic OR (hazard AND (physical AND chemical)) OR (work AND (night OR day)) OR (occupation AND stress) OR (work AND characteristics))

AND ((disease AND management) OR (health AND circles) OR amigo OR (primary AND intervention) OR “prima-ef” OR (health AND management) OR (workplace AND health) OR (health AND promotion) OR (shift AND work) OR risk management OR self-scheduling OR (flexible AND scheduling) OR (work AND schedule) OR (reconciling AND work) OR (family AND life) OR (“return-to” AND work) OR (flexible AND work) OR flexibility OR (compressed AND (hour OR work OR week)) OR (flexible AND salary) OR (life AND balance) OR (work AND (life OR balance)) OR employee OR employer OR (quality AND life) OR (cognitive AND (therapy OR behavioural)))

AND “intervention*”
