# Supplementary material for: Effects of organisational-level interventions at work on employees’ health: a systematic review
Source: BMC Public Health. 2014 Feb 8;14:135. doi: 10.1186/1471-2458-14-135 (PMC3929163; doi:10.1186/1471-2458-14-135)
Supplement: Additional file 2 — Results Tables. [file 1471-2458-14-135-S2.doc]

# Effects of organisational-level interventions at work on employees’ health: A systematic review

Diego Montano^1*^, Hanno Hoven^1,2^, Johannes Siegrist^1^

*^1^ Senior Professorship “Work Stress Research”, Faculty of Medicine, Duesseldorf University*

*^2^Institute of Medical Sociology, Faculty of Medicine, Duesseldorf University*

*Correspondence: [diego.montano@med.uni-duesseldorf.de](mailto:diego.montano@med.uni-duesseldorf.de)

# Appendix 2. Supplemented Tables S1 and S2

Table S1. Detailed description of studies reviewed.

Identifier = First author and publication year. Designs: RCT = Randomized controlled trial, QE = Quasi-experimental, QE prospective = Quasi-experimental with control group. NA = not available or not applicable.

| **Identifier** | **Year** | **n basel.** | **n follow** | **Follow up** | **n control** | **Occupation** | **Country** | **EGP** | **Design** | **Quality** | **Evidence** | **Effect** | **Intervention type** | **Working conditions changed** |
| --- | --- | --- | --- | --- | --- | --- | --- | --- | --- | --- | --- | --- | --- | --- |
| Morken 2002 [1] | 2002 | 558 | 414 | 1,3 | 35 | Aluminium workers | Norway | VI-VII | RCT | 11 | high | no | Participatory | Material, Organisation |
| Mygind 2006 [2] | 2006 | 644 | 495 | 1 | 280 | Slaughterhouse workers | Denmark | VI-VII | RCT | 9 | high | yes | Participatory | Material, Organisation |
| Oude Hengel 2012 [3] | 2012 | 293 | 213 | 1 | 122 | Construction workers | Denmark | VI-VII | RCT | 9 | high | no | Other | Time |
| Schrijnemaekers 2003 [4] | 2003 | 300 | 242 | 1 | 145 | Health care workers | The Netherlands | I-III | RCT | 9 | high | no | Other | Organisation |
| Boggild 2001 [5] | 2001 | 172 | 101 | 1 | 60 | Health care workers | Denmark | I-III | QE prospective | 3 | medium | yes | Shift schedules | Time |
| Bond 2001 [6] | 2001 | 97 | 53 | 1 | 49 | Civil servants | UK | I-III | QE prospective | 3 | medium | no | Participatory | Organisation |
| Bourbonnais 2006 [7] | 2006 | 492 | 302 | 1 | 311 | Health care workers | Canada | I-III | QE prospective | 8 | medium | yes | Participatory | Material, Time, Organisation |
| Bourbonnais 2011 [8] | 2011 | 492 | 247 | 3 | 220 | Health care workers | Canada | I-III | QE prospective | 7 | medium | yes | Participatory | Material, Time, Organisation |
| Carrivick 2002 [9] | 2002 | 507 | 265 | 3 | 128 | Cleaners | Australia | VI-VII | QE prospective | 7 | medium | yes | OI | Material, Time, Organisation |
| Dahl-Jorgensen 2005 [10] | 2005 | 415 | 282 | 3 | 139 | Health care workers / Retail workers | Norway | I-III, VI-VII | QE prospective | 8 | medium | no | Participatory | Organisation |
| DeJoy 2010 [11] | 2010 | 2207 | 1510 | 2 | NA | Retail workers | USA | I-III, VI-VII | QE prospective | 8 | medium | yes | Participatory | Time, Organisation |
| Evans 1999 [12] | 1999 | 41 | 36 | 1 | 31 | Bus drivers | Sweden | VI-VII | QE prospective | 3 | medium | yes | OI | Organisation |
| Garde 2012 [13] | 2012 | 299 | 297 | 1 | 183 | Health care workers | Denmark | I-III | QE prospective | 7 | medium | no | Shift schedules | Time |
| Garde 2011 [14] | 2011 | 840 | 791 | 1 | 347 | Health care and call center workers | Denmark | I-III, VI-VII | QE prospective | 11 | medium | yes | Shift schedules | Time |
| Kawakami 1997 [15] | 1997 | 285 | 187 | 2 | 175 | Machine operators and technicians | Japan | VI-VII | QE prospective | 5 | medium | yes | Other | Material, Organisation |
| Laing 2007 [16] | 2007 | 81 | 82 | 0,9 | 35 | Manufacture workers | Canada | VI-VII | QE prospective | 3 | medium | no | Participatory | Material, Organisation |
| Lemstra 2006 [17] | 2006 | 470 | 0 | 2 | 185 | Meat industry workers | Canada | VI-VII | QE prospective and retrospective | 5 | medium | yes | Other | Material, Organisation |
| Logan 2005 [18] | 2005 | 67 | 64 | 0,7 | 33 | Project managers | USA | I-III | RCT | 3 | medium | no | OI | Organisation |
| Mattila 2006 [19] | 2006 | 525 | 420 | 2 | 272 | Maintenance and construction workers | Finland | VI-VII | QE prospective | 11 | medium | no | Participatory | Organisation |
| Melchior 1996 [20] | 1996 | 361 | 161 | 2,5 | 101 | Health care workers | The Netherlands | I-III | QE prospective | 6 | medium | no | Other | Organisation |
| Michie 2004 [21] | 2004 | 312 | 0 | 1 | 91 | Cleaners | USA | VI-VII | QE prospective and retrospective | 5 | medium | no | OI | Time, Organisation |
| Mikkelsen 2000 [22] | 2000 | 135 | 59 | 0,25 | 71 | Health care workers | Norway | I-III | QE prospective | 3 | medium | no | Participatory | Organisation |
| Mikkelsen 1999 [23] | 1999 | 162 | 125 | 1 | 70 | Postal office employees | Norway | I-III | QE prospective | 7 | medium | no | Participatory | Organisation |
| Nabe-Nielsen 2011 [24] | 2011 | 309 | 297 | 1 | 187 | Health care workers | Denmark | I-III | QE prospective | 9 | medium | yes | Shift schedules | Organisation |
| Pryce 2006 [25] | 2006 | 177 | 166 | 1,7 | 91 | Health care workers | Denmark | I-III | QE prospective | 5 | medium | no | Shift schedules | Time |
| Rasmussen 2006 [26] | 2006 | 620 | 480 | 3,5 | 270 | Manufacture workers | Denmark | VI-VII | QE prospective | 9 | medium | yes | Participatory | Material, Organisation |
| Reynolds 1997 [27] | 1997 | 119 | 112 | 1 | 43 | Civil servants | UK | I-III, VI-VII | QE prospective | 4 | medium | no | Other | Organisation |
| Smedley 2003 [28] | 2003 | 1239 | 1078 | 2,7 | 340 | Health care workers | UK | I-III | QE prospective | 9 | medium | no | Other | Material |
| Tsutsumi 2009 [29] | 2009 | 97 | 77 | 1 | 50 | Assembly line workers | Japan | VI-VII | RCT | 4 | medium | yes | Participatory | Organisation |
| Andersen 2010 [30] | 2010 | 1024 | 1736 | 5 | NA | Human service sector workers | Denmark | I-III | QE one-group prospective | 10 | low | no | OI | Organisation |
| Anderzen 2005 [31] | 2005 | 303 | 303 | 1 | NA | Civil servants | Sweden | I-III | QE one-group prospective | 8 | low | yes | OI | Organisation |
| Cole 2009 [32] | 2009 | 157 | 157 | 1 | 101 | Courier depots, auto parts, and manufacturing workers | Canada | VI-VII | QE cross-sectional | 3 | low | no | Participatory | Material, Organisation |
| Collins 2004 [33] | 2004 | 1728 | 0 | 6 | NA | Health care workers | USA | I-III | QE one-group prospective | 4 | low | yes | OI | Material, Organisation |
| Elke 2005 [34] | 2005 | 216 | 185 | 2 | NA | Manufacture workers | Germany | I-III, VI-VII | QE one-group prospective | 2 | low | no | OI | Material, Organisation |
| Evanoff 1999 [35] | 1999 | 67 | 87 | 1,25 | NA | Health care workers | USA | I-III | QE one-group prospective | 1 | low | yes | Participatory | Material |
| Heaney 1993 [36] | 1993 | 176 | 0 | 5 | NA | Manufacture workers | USA | VI-VII | QE one-group prospective | 5 | low | no | Participatory | Organisation |
| Petterson 1998 [37] | 1998 | 3506 | 2617 | 1 | NA | Health care workers | Sweden | I-III | QE one-group prospective | 8 | low | no | Participatory | Organisation |
| Porru.2011 [38] | 2011 | 298 | 248 | 13 | NA | Foundry workers | Italy | VI-VII | QE one-group prospective | 7 | low | yes | OI | Material, Organisation |
| Wahlstedt 1997 [39] | 1997 | 136 | 100 | 1 | NA | Postal workers | Sweden | I-III, VI-VII | QE one-group prospective | 4 | low | no | Participatory | Time, Organisation |

Table S2. Detailed description of implementation and results. Identifier = First author and publication year.

| **Identifier** | **Implementation** | **Outcomes** | **Measures** | **Results** |
| --- | --- | --- | --- | --- |
| Morken 2002 [1] | Employees formed groups, discussed ergonomic risks and the conditions for an optimal work environment at the technical and organisational level. The groups were assisted by physiotherapists. Solution plans were proposed and integrated into the internal control system for health and safety in the plant. | MSS | Scandinavian Nordic Questionnaire (SNQ) | No significant effects of intervention on selected health outcomes |
| Mygind 2006 [2] | (1) Top-down. A management system focusing on skin risk is established. (2) Bottom-up. A group of employees is trained to identify risk factors and to implement risk control. | Skin health | Upper extremities eczema | 32% overall eczema frequency reduction |
| Oude Hengel 2012 [3] | Intervention combines theoretical information with practical information from stakeholders. The intervention consisted of (1) physical training sessions, and (2) a Rest-Break tool for reducing fatigue. | General health | Need for recovery | No significant effects of intervention on selected health outcomes |
| Schrijnemaekers 2003 [4] | Introduction of an alternative care therapy and its effects on professional caregivers of disoriented elderly. The intervention offered consisted of three successive elements: clinical lessons, a training program, and supervision meetings spread over a total period of 8 months | Burnout | Emotional exhaustion, depersonalization, personal accomplishment | No significant effects of intervention on selected health outcomes. Control group improved significantly on the scale of personal accomplishment (difference: 1.46, p < 0.5) |
| Boggild 2001 [5] | Shift schedules | Ischemic heart disease risk | Lipoproteins | Difference HDL (mmol/L) 0.1, P=0.004, LDL (mmol/L) -0.2, p=0.005 |
| Bond 2001 [6] | Participative action research intervention. A steering committee was created in order to develop and implement work organization changes that might increase employees' job control and, thereby, improve the stress-related variables in their units. An action plan was developed in the following problem areas: assignment distribution procedures, within-unit consultation and communication, and informal performance feedback. | Mental health, sickness absence | Mental health, sickness absence | Group x time significant effects for selected health outcomes at follow up in ANOVA analyses (F = 8.60, df=1,50; F = 4.26, df=1, 87, resp.). |
| Bourbonnais 2006 [7] | Factors affecting psychosocial conditions were identified by an intervention team, and by quantitative and qualitative analysis. Changes were made regarding (i) team work and spirit, (ii) staffing processes, (iii) work organization, (iv) training, (v) communication, and (vi) ergonomics. | Burnout | Burnout | No statistically significant differences between groups for burnout: -0.65 [-2.33, 1.02]. |
| Bourbonnais 2011 [8] | Factors affecting psychosocial conditions were identified by an intervention team, and by quantitative and qualitative analysis. Changes were made regarding (i) team work and spirit, (ii) staffing processes, (iii) work organization, (iv) training, (v) communication, and (vi) ergonomics. | Burnout | Burnout | Differences : Burnout: -3.1 [-5.1, -1.1]. |
| Carrivick 2002 [9] | Iterative injury risk identification, assessment, and control process intervention by creation of a workplace assessment team of cleaning service staff, supervisors and an ergonomist. Various hazard controls were implemented: change of purchasing procedures, safer cleaning equipment and machinery, increased rate of daily rotation to reduce repetitive strain. | Injuries | Lost time injury (LTI) | The OR in the intervention group for lost time injury was 0.35 [0.23, 0.55]. In the control group OR 1.54 [1.17, 2.00] |
| Dahl-Jorgensen 2005 [10] | (1) Identification of factors perceived by employees as causes of stress at work, (2) emphasizing job redesign / environmental causes of disease, and (3) active involvement of employees for the improvement of the organization as a system. | Stress, somatic symptoms, burnout, and absenteeism | Copper's Job Stress Scale, self-reported health, Burnout Inventory, sick days. | There were neither intervention effects nor time x group effects in MANOVA analyses (F(1, 321) = 1.50, p=0.091, and F(1, 321)=1.12, p=0.322, resp.). ANOVA analyses were not significant. |
| DeJoy 2010 [11] | Creation of a problem solving team of employees. The intervention team was broadly representative of the employee mix at each location. Teams were charged with developing, implementing, and evaluating tailored plans of action for addressing the issues or problems identified within their stores. | Employee health and wellbeing | Perceived health, perceived safety at work, alcohol use, high risk health behaviors, preventive health behaviors | Treatment-by-change interactions of employee health: t[17] = 2.07, p < 0.04, eta^2=0.01. Interactions for alcohol use, engaging in high risk health behaviors, and preventive health measures were not statistically significant. |
| Evans 1999 [12] | Construction of separate bus lanes for the most congested parts of the bus routes, reconfiguration of parts of the route to minimize difficult turns, construction of passenger peninsulas, installation of a traffic signal priority system for the bus. | Blood pressure, heart rate | Blood pressure, heart rate | Changes in job hassles were significantly correlated to systolic blood pressure (r = 0.27, p < 0.05), and heart rate (r = 0.26, p < 0.05). |
| Garde 2012 [13] | Multisite intervention. Three workplaces introduced self-rostering by use of an IT-software. In four workplaces employees were given influence on a fixed rota schedule. | Sleep quality | Karolinska Sleep Questionnaire | No overall effects on the measures of sleep quality |
| Garde 2011 [14] | Self-rostering by use of commercially available IT software chosen by the intervention companies. Three intervention types analyzed. Intervention A included a time bank and the possibility to choose separately starting time and length of shift. Intervention B did not include a time bank, and it was only possible to choose between a number of predefined types of duties. Intervention C included a time bank, but it was still only possible to choose between a number of predefined duties. Intervention C did not require 24-hour staffing. | Sleep quality, need for recovery, mental distress and somatic symptoms | Karolinska Sleep Questionnaire (KSQ), awakening index (AWI), Symptom Checklist 90 (SCL-90) | Need for recovery decreased in groups A and B (adj. mean=-0.17 [-0.29, -0.04], adj. mean=-0.17 [-0.27, -0.07], resp.). In group B there were fewer symptomatic symptoms (adj. mean=-0.10 [-0.19, -0.02], less mental distress (adj. mean=-0.13 [-0.23, -0.03], and better sleep (adj. mean=0.17 [0.04, 0.30]. |
| Kawakami 1997 [15] | Creation of an intervention committee. Only the supervisors identified stressors. Following changes were made in order to reduce stress levels of employees: (1') Machine performance improved, (2) reduction of checkpoints assessments, (3) training of employees in new technologies, (4) placing of additional supervisors in order to facilitate communication between management and employees. | Depressive symptoms, sick leave, blood pressure | Zung's Depression Scores, self-reported sick leaves, systolic and diastolic blood pressure. | Statistically significant treatment effect (ANCOVA) F=4.96, df=2, p = 0.0025) for males. Significant reduction of sick-leave length (chi=18.4, df=2). No changes in blood pressure. |
| Laing 2007 [16] | Nine participatory psychosocial activities were implemented: (1) ergonomics newsletter, (2) ergonomics bulletin board with relevant information about the intervention, (3) installation of an ergonomics suggestion box for employees, (4) Description of the program in a corporate newsletter, (5) ergonomics presentations at shift meetings, (6) logo contest for the ergonomics program, (7) creation of ergonomics committees with representation of employees, (8) trials of ergonomics measures in workstations, and (9) employees survey. | MSS | Two pain-related items | No significant time x intervention effects for back, shoulder/upper arm, forearm/hand, leg/lower limb pain (p = 0.973, 0.356, 0.286, 0.530, resp.) |
| Lemstra 2006 [17] | (1) Changes in worker rotation schedules, (2) reduction of lifting loads, (3) ergonomic redesign of tasks | Work related back pain injuries | Injury claims | Significant reductions of back-pain time-loss days. Rate ratio = 0.02, [0.01-0.04]. |
| Logan 2005 [18] | (1) Managers had more control over service providers selection, (2) introduction of additional night time road service dispatchers, (3) training on financial reporting | Somatic complaints, depressive symptoms | Center for Epidemiologic Studies Depression Scale (CES-D), Somatic Complaints Scale | The intervention had no effects neither on control nor stress-related outcomes. |
| Mattila 2006 [19] | Employees participated in a two-session participative work-conference in order to (1) create visions of well-being, (2) recognize obstacles to fulfilling these visions, (3) set goals for developing the psychosocial work environment and well-being, and (4) make a practical development plan for the work unit. | Burnout | Maslach Burnout Inventory | No significant treatment x group effects of intervention on selected health outcomes (ANCOVA) |
| Melchior 1996 [20] | Introduction of an innovated nursing care delivery with a special focus on primary nursing. | Burnout | Emotional exhaustion, depersonalization, personal accomplishment | There were no significant differences of MANCOVA and ANOVA analysis with pretest results as covariates. |
| Michie 2004 [21] | Introduction of a system to report problems and request help, management consultation with cleaning staff over new uniform, agreement that time for changing into and out of the uniform be within work time, funding of an annual Christmas party, and giving a choice of timing of the morning and lunch breaks. | Sickness absence | Sickness absence rate | There were no intervention effects at 1-year follow-up regarding absence rates (t = -0.249, df=23, p = 0.808). |
| Mikkelsen 2000 [22] | Employees identify settings that need to be improved, create work groups for each setting, define an implementation plan, evaluate the results of the 12-week intervention and suggest further developments. | Self-reported health | Health Inventory (HUI) | No significant effects of intervention on selected health outcomes |
| Mikkelsen 1999 [23] | Supervisors and managers were involved. Participants formulated what they believed to be the ideal model organization, formulated their complaints, and designed alternatives and improvements. Cause-and-effect relationships were discussed and problems identified.. Vertical and horizontal control was attempted through steering committees with representatives from supervisors, management, and union. | Subjective health | Self-reported health | No significant effects of intervention on selected health outcomes |
| Nabe-Nielsen 2011 [24] | Work schedule three-arm intervention. Intervention group A: computer assisted self-scheduling. Intervention B and C fixed, rotating schedule. | Cardiovascular risk factors | Waist and hip circumference, total cholesterol, HDL cholesterol, HbA1c | Time x group significant effects in intervention B for stress score and HbA1c (difference: -0.43 [-0.69, -0.17], and OR 1.04 [1.01, 1.07], resp.) |
| Pryce 2006 [25] | Steering committees composed of safety, trade, union and project representatives were created to identify the appropriate schedule intervention to be implemented. An open-rota system was implemented, in which employees were asked to schedule their shift preferences. | Global self-rated health | Self-rated health | No intervention effects in evaluated health outcomes (MANOVA) |
| Rasmussen 2006 [26] | Intervention based on participatory action research. Groups were created to work on following areas: (1) chemical products, (2) personal protective devices, (3) education and learning, (4) proactive safety organization, (5) information and communication, (6) accident and injury registration system, (7) safety rounds, (8) creation of teams responsible for quality, safety, etc. | Eczema, accidents and injuries | Eczema, injury and accident rates, Job Content Questionnaire | Eczema incidence in both groups significantly decreased (in average from 68 cases per year/1000 employees to 10-12 cases per year/1000 employees).. In the intervention group accidents decreased significantly. |
| Reynolds 1997 [27] | The intervention consisted of an individual and an organisational intervention. For the latter, it was aimed to increase the opportunity and level of participation and control of employees in the day to day decisions within their work teams and unit. Additional aims were to clarify responsibilities, to increase the level of job-related information employees received, and to enable supervisors to give clear feedback about performance. | Well-being | Psychological and physical symptoms | There were no intervention effects regarding the organisational level intervention. Mean levels of physical symptoms even increased. |
| Smedley 2003 [28] | (1) Revision of the hospital-wide manual handling policy, (2) awareness sessions with the Health and Safety Advisory Committee, (3) dissemination of new policy to all members of the staff, (4) purchase of new sliding sheets and additional lifting and handling equipment, (5) identification of areas with high job-related low-back pain, (6) establishment of a network of ward-based \link nurses\" to facilitate the intervention throughout the organization" | Low back pain | One-month prevalence of low-back pain | No significant changes in the prevalence of low-back pain in the intervention group. |
| Tsutsumi 2009 [29] | (1) Building on local practice, (2) focusing on achievements (good practices, etc.), (3) linking working conditions to management goals, (4) encouraging exchange of experience, (5) promoting employee involvement, and (5) learning-by-doing. | Mental health | General health questionnaire | Time x group significant effects for general health (F = 6.23, p =0.014) |
| Andersen 2010 [30] | Changes of the decision making, information systems. Merging of departments, building of new teams with the aim of increasing influence and engagement. | Burnout | Burnout scores | Difference 4.66 [2.0 , 7.2] |
| Anderzen 2005 [31] | Enhancement in the following areas: leadership, participatory management, employeeship, management performance feedback, work-related exhaustion. Managers and employees made their own action and implementation plan. | Employee well-being, self-rated health, absenteeism | Well-being, lipids, stress-related hormones, sickness, absenteeism | Well-being: Before: 68 (SD=8.6) , After: 72 (SD=6.7), P(time) < 0.05. Self-rated health: Before: 3.7 (SD=0.35) , After: 3.8 (SD=0.41), P(time) > 0.05. Cortisol (nmol/L): Before: 376 (SD=47.1) , After: 433 (SD=82.3), P(time) < 0.001 |
| Cole 2009 [32] | Participatory ergonomics intervention consisting of multiple implementations. Multisite intervention. Among others: implementing plan wide intervention program, workplace changes, reduction of workers' physical demands. | MSS, pain | Pain | Changes across all sites: Pain: 0.02 (SD=0.80). |
| Collins 2004 [33] | The intervention included mechanical lifting equipment, repositioning aids, worker training on the use of lifts, and a written zero-lift policy. | Work related back pain injuries | Injury records | Significant injury reduction, Rate ratio = 0.65 [0.50, 0.73] |
| Andersen 2010 [30] | Integrated occupational health and safety management system. Implementation at the management, human resources, information, and work station levels. | Injuries and absenteeism | Injury rate and disability days | 50% reduction of injury rates after one year. In one group, however, injury rates increased after 2 years. Disability days reduction was not achieved. |
| Anderzen 2005 [31] | A participatory ergonomics team was created in order to identify and prioritize safety problems and to evaluate and implement possible solutions. Primary interventions were the development of standardized lifting techniques and the training of all orderlies in the use of these procedures. | Injuries and absenteeism | OSHA logs | Adjusted risk ratio (RR) of recordable injury: 0.64 [0.44, 0.93]. RR of disability day 0.40 [0.21, 0.75]. Significant reductions of self-reported neck, shoulders, and back pain frequencies. |
| Cole 2009 [32] | Creation of a Stress and Wellness Committee (SWC) representative of the workforce. Solutions were proposed to following problem areas: lack of information, communication, and feedback. | Depressive symptoms | Center for Epidemiologic Studies Depression Scale (CES-D) | Involvement in the intervention was associated with increases of symptoms in one plant and decreases in other. |
| Petterson 1998 [37] | Organisational intervention program in the following areas: organisational efficiency, skills development, workload and social climate. | Psychosomatic symptoms | Self-reported psychosomatic symptoms, exhaustion | No intervention effects in evaluated health outcomes |
| Porru.2011 [38] | Multicomponent intervention. Components: (1) Institution of a prevention team of all stakeholders for reducing occupational injuries (OI), (2) periodical team meetings, (3) selected technical and organisational changes, (4) first-aid team organization, (5) education of managers, supervisors and workers on safety, (6) health surveillance | Injuries | Injury rate | Reduction of incidence rates between 51% and 57%, p < 0.001) |
| Wahlstedt 1997 [39] | (1) Two separate production areas were formed. The role of management in each area was clarified and production goals stated more precisely. The number of supervisors decreased. It was aimed to increase the influence of the staff on the work situation. (2) The number of intermediate supervisors was reduced, in order to allow them more work control. (3) The work force was increased by 2.5% in order to handle periods of excessive work load. (4) The information system was improved through weekly staff meetings and by keeping notice-boards up to date. (5) The shift system was profoundly changed, a shift of 3 to 4 weeks was introduced, and the meal breaks became more regular. (6) An automatic food vending machine and microwave ovens were introduced. | Psychosomatic complaints | Gastrointestinal complaints and sleep difficulties | Significant increases of sleep difficulties from 10.8 to 11.6 (p < 0.05). The number of gastrointestinal complaints did not differ significantly. |

**References**

1. Morken T, Moen B, Riise T, Hauge SH, Holien S, Langedrag A, Olson H, Pedersen S, Saue IL, Seljebo GM, Thoppil V: **Effects of a training program to improve musculoskeletal health among industrial workers - Effects of supervisors role in the intervention**. *Int J Ind Ergonom* 2002, **30**:115-127.

2. Mygind K, Borg V, Flyvholm M, Sell L, Jepsen KF: **A study of the implementation process of an intervention to prevent work-related skin problems in wet-work occupations**. *Int Arch Occup Environ Health* 2006, **79**:66-74.

3. Oude Hengel KM, Blatter BM, Joling CI, van der Beek AJ, Bongers PM: **Effectiveness of an intervention at construction worksites on work engagement, social support, physical workload, and need for recovery: results from a cluster randomized controlled trial**. *BMC Public Health* 2012, **12**:1008.

4. Schrijnemaekers VJJ, van Rossum E, Candel MJJM, Frederiks CMA, Derix MMA, Sielhorst H, van den Brandt PA: **Effects of Emotion-Oriented Care on Work-Related Outcomes of Professional Caregivers in Homes for Elderly Persons**. *J Gerontol B-Psychol* 2003, **58**:S50.

5. Boggild H, Jeppesen HJ: **Intervention in shift scheduling and changes in biomarkers of heart disease in hospital wards**. *Scand J Work Environ Health* 2001, **27**:87-96.

6. Bond FW, Bunce D: **Job control mediates change in a work reorganization intervention for stress reduction**. *J Occup Health Psychol* 2001, **6**:290-302.

7. Bourbonnais R, Brisson C, Vinet A, Vezina M, Abdous B, Gaudet M: **Effectiveness of a participative intervention on psychosocial work factors to prevent mental health problems in a hospital setting**. *Occup Environ Med* 2006, **63**:335-342.

8. Bourbonnais R, Brisson C, Vézina M: **Long-term effects of an intervention on psychosocial work factors among healthcare professionals in a hospital setting**. *Occup Environ Med* 2011, **68**:479-486.

9. Carrivick PJW, Lee AH, Yau KKW: **Effectiveness of a workplace risk assessment team in reducing the rate, cost, and duration of occupational injury**. *J Occup Environ Med* 2002, **44**:155-159.

10. Dahl-Jorgensen C, Saksvik PO: **The impact of two organizational interventions on the health of service sector workers**. *Int J Health Serv* 2005, **35**:529-549.

11. DeJoy DM, Wilson MG, Vandenberg RJ, McGrath-Higgins AL, Griffin-Blake CS: **Assessing the impact of healthy work organization intervention**. *J Occup Organ Psych* 2010, **83**:139-165.

12. Evans GW, Johansson G, Rydstedt L: **Hassles on the job: A study of a job intervention with urban bus drivers**. *J Organ Behav* 1999, **20**:199.

13. Garde AH, Albertsen K, Nabe-Nielsen K, Carneiro IG, Skotte J, Hansen SM, Lund H, Hvid H, Hansen AM: **Implementation of self-rostering (the PRIO project): Effects on working hours, recovery, and health**. *Scand J Work Environ Health* 2012, **38**:314-326.

14. Garde AH, Nabe-Nielsen K, Aust B: **Influence on working hours among shift workers and effects on sleep quality - An intervention study**. *Applied Ergonomics* 2011, **42**:238-243.

15. Kawakami N, Araki S, Kawashima M, Masumoto T, Hayashi T: **Effects of work-related stress reduction on depressive symptoms among Japanese blue-collar workers**. *Scand J Work Environ Health* 1997, **23**:54-59.

16. Laing AC, Cole DC, Theberge N, Wells RP, Kerr MS, Frazer MB: **Effectiveness of a participatory ergonomics intervention in improving communication and psychosocial exposures**. *Ergonomics* 2007, **50**:1092-1109.

17. Lemstra M, Olszynski WP: **The effectiveness of standard care, early intervention, and occupational management in worker's compensation claims**. *Spine* 2003, **28**:299-304.

18. Logan MS, Ganster DC: **An Experimental Evaluation of a Control Intervention to Alleviate Job-Related Stress**. *J Manage* 2005, **31**:90-107.

19. Mattila P, Elo A, Kuosma E, Kylä-Setälä E: **Effect of a participative work conference on psychosocial work environment and well-being**. *Eur J Work Organ Psy* 2006, **15**:459-476.

20. Melchior ME, Philipsen H, Abu-Saad HH, Halfens RJ, van de Berg AA, Gassman P: **The effectiveness of primary nursing on burnout among psychiatric nurses in long-stay settings**. *J Adv Nurs* 1996, **24**:694-702.

21. Michie S, Wren B, Williams S: **Reducing absenteeism in hospital cleaning staff: pilot of a theory based intervention**. *Occup Environ Med* 2004, **61**:345-349.

22. Mikkelsen A, Saksvik PØ, Landsbergis P: **The impact of a participatory organizational intervention on job stress in community health care institutions**. *Work and Stress* 2000, **14**:156-170.

23. Mikkélsen A, Saksvik PO: **Impact of a participatory organizational intervention on job characteristics and job stress**. *Int J Health Serv* 1999, **29**:871-893.

24. Nabe-Nielsen K, Garde AH, Diderichsen F: **The effect of work-time influence on health and well-being: A quasi-experimental intervention study among eldercare workers**. *Int Arch Occup Environ Health* 2011, **84**:683-695.

25. Pryce J, Albertsen K, Nielsen K: **Evaluation of an open-rota system in a Danish psychiatric hospital: A mechanism for improving job satisfaction and work-life balance**. *J Nurs Manag* 2006, **14**:282-288.

26. Rasmussen K, Glasscock DJ, Hansen ON, Carstensen O, Jepsen JF, Nielsen KJ: **Worker participation in change processes in a Danish industrial setting**. *Am J Ind Med* 2006, **49**:767-779.

27. Reynolds S: **Psychological well-being at work: Is prevention better than cure?** *J Psychosom Res* 1997, **43**:93-102.

28. Smedley J, Trevelyan F, Inskip H, Buckle P, Cooper C, Coggon D: **Impact of ergonomic intervention on back pain among nurses**. *Scand J Work Environ Health* 2003, **29**:117-123.

29. Tsutsumi A, Nagami M, Yoshikawa T, Kogi K, Kawakami N: **Participatory intervention for workplace improvements on mental health and job performance among blue-collar workers: a cluster randomized controlled trial**. *J Occup Environ Med* 2009, **51**:554-563.

30. Andersen I, Borritz M, Christensen KB, Diderichsen F: **Changing job-related burnout after intervention--a quasi-experimental study in six human service organizations**. *J Occup Environ Med* 2010, **52**:318-323.

31. Anderzén I, Arnetz BB: **The impact of a prospective survey-based workplace intervention program on employee health, biologic stress markers, and organizational productivity**. *J Occup Environ Med* 2005, **47**:671-682.

32. Cole DC, Theberge N, Dixon SM, Rivilis I, Neumann WP, Wells R: **Reflecting on a program of participatory ergonomics interventions: A multiple case study**. *Work* 2009, **34**:161-178.

33. Collins JW, Wolf L, Bell J, Evanoff B: **An evaluation of a "best practices" musculoskeletal injury prevention program in nursing homes**. *Inj Prev* 2004, **10**:206-211.

34. Elke G, Zimolong B: **An intervention study of the impact of human resource management concerning occupational health and safety**. *Z Arb Organ* 2005, **49**:117-130.

35. Evanoff BA, Bohr PC, Wolf LD: **Effects of a participatory ergonomics team among hospital orderlies**. *Am J Ind Med* 1999, **35**:358-365.

36. Heaney CA, Israel BA, Schurman SJ, Baker EA, House JS, Hugentobler M: **Industrial Relations, Worksite Stress Reduction, and Employee Well-Being: A Participatory Action Research Investigation**. *J Organ Behav* 1993, **14**:495-510.

37. Petterson I, Arnetz BB: **Psychosocial stressors and well-being in health care workers. The impact of an intervention program**. *Soc Sci Med* 1998, **47**:1763-1772.

38. Porru S, Calza S, Arici C: **An effectiveness evaluation of a multifaceted preventive intervention on occupational injuries in foundries: a 13-year follow-up study with interrupted time series analysis**. *Int Arch Occup Environ Health* 2011, **84**:867-876.

39. Wahlstedt KGI, Edling C: **Organizational changes at a postal sorting terminal—their effects upon work satisfaction, psychosomatic complaints and sick leave**. *Work & Stress* 1997, **11**:279-291.
